# Negative regulation of SH2B3 by SMYD5 controls epithelial-mesenchymal transition in lung cancer

**DOI:** 10.1016/j.mocell.2024.100067

**Published:** 2024-05-07

**Authors:** In Hwan Tae, Tae Young Ryu, Yunsang Kang, Jinkwon Lee, Kwanho Kim, Jeong Min Lee, Hee-Won Kim, Jung Heon Ko, Dae-Soo Kim, Mi-Young Son, Hyun-Soo Cho

**Affiliations:** 1 Stem Cell Convergence Research Center, Korea Research Institute of Bioscience and Biotechnology, Daejeon 34141, Republic of Korea; 2 Functional Genomics, Korea University of Science and Technology, Daejeon 34113, Republic of Korea; 3 Department of Biological Science, Sungkyunkwan University, Suwon 16419, Republic of Korea

**Keywords:** Epithelial-mesenchymal transition, Lung cancer, Metastasis, SMYD5

## Abstract

The main cause of death in lung cancer patients is metastasis. Thus, efforts to suppress micrometastasis or distant metastasis in lung cancer, identify therapeutic targets and develop related drugs are ongoing. In this study, we identified SET and MYND domain-containing protein 5 (SMYD5) as a novel metastasis regulator in lung cancer and found that SMYD5 was overexpressed in lung cancer based on both RNA-sequencing analysis results derived from the TCGA portal and immunohistochemical analysis results; knockdown of SMYD5 inhibited cell migration and invasion by changing epithelial-mesenchymal transition markers and MMP9 expression in NCI-H1299 and H1703 cell lines. Additionally, SMYD5 knockdown increased Src homology 2-b3 expression by decreasing the level of H4K20 trimethylation. Furthermore, in an in vitro epithelial-mesenchymal transition system using TGF-β treatment, SMYD5 knockdown resulted in reduced cell migration and invasion in the highly invasive NCI-H1299 and H1703 cell lines. Based on these findings, we propose that SMYD5 could serve as a potential therapeutic target for lung cancer treatment and that cotreatment with an SMYD5 inhibitor and chemotherapy may enhance the therapeutic effect of lung cancer treatment.

## INTRODUCTION

Metastasis is a primary contributor to mortality in lung cancer patients. The liver, nervous system, and bone are the most common sites of metastasis in lung cancer, and liver metastasis is associated with the poorest prognosis (Riihimaki et al., 2014). Despite the administration of chemotherapy or antibody therapy for lung cancer treatment, the occurrence of micrometastasis and distant metastasis remains a challenge. Consequently, inhibiting lung cancer metastasis is a crucial objective for enhancing the efficacy of chemotherapy. However, there is a persistent need for the development and validation of targets to effectively inhibit lung cancer metastasis.

Epigenetic modifiers, including histone methyltransferases, deoxyribo nucleic acid (DNA) methyltransferases, and mirco ribonucleic acid (miRNA)s, have been clearly linked to cancer progression in various types of cancer (Michalak et al., 2019). Recently, the Enhancer of zeste homolog 2 (EZH2) inhibitor tazemetostat received U.S. Food and Drug Administration (FDA) approval for the treatment of patients with solid malignancies and hematologic cancers (Straining and Eighmy, 2022). Overexpression of histone methyltransferases has been observed in multiple cancer types and is known to play a crucial role in cancer progression and metastasis (Kim et al., 2023, Kim et al., 2022, Kim et al., 2020, Lee et al., 2021, Ryu et al., 2022). Thus, histone methyltransferase was recognized as a novel therapeutic target for cancer treatment (Liu and Wang, 2016).

SET (Su(var)3-9, enhancer-of-zeste and trithorax) and MYND (myeloid, nervy, and DEAF-1) domain-containing protein 5 (SMYD5) is a methyltransferase for the methylation of histone H3 lysine 36 (H3K36; Aljazi et al., 2022, Zhang et al., 2022) and 37 (Aljazi et al., 2022), and histone H4 lysine 20 (H4K20; Kidder et al., 2017b). In the embryonic stem (ES) cell self-renewal process, depletion of SMYD5 dysregulated Octamer-4 (OCT4) expression via a decrease in H4K20 trimethylation (Kidder et al., 2017b). Additionally, during embryogenesis in zebrafish, Smyd5 plays an important role in hematopoiesis (Fujii et al., 2016), and SMYD5 controls chromosome integrity in ES differentiation (Kidder et al., 2017a). Moreover, in the immune system, SMYD5 may negatively regulate the macrophage inflammatory response (Rubio-Tomas, 2021), and SMYD5 facilitates HIV-1 transcription as a host activator in primary T cells (Boehm et al., 2023). In cancers, SMYD5 is overexpressed in gastric cancer (Reyes et al., 2021) and hepatocellular carcinoma. In hepatocellular carcinoma cell lines, SMYD5 knockdown suppressed cell migration and invasion and enhanced paclitaxel sensitivity (Chi et al., 2022). However, although SMYD5 is related to cancer progression and other biological processes, the functions of SMYD5 in cancer cell proliferation and metastasis are unclear.

Src homology 2-b3 (SH2B3, also called lymphocyte adapter protein; LNK) is a member of the SH2B family and is an adaptor protein for the regulation of signaling pathways and multiple tyrosine kinases (Dale and Madhur, 2016). In cancers, overexpression of SH2B3 reduced the invasion ability in HCT116 and HT29 colon cancer cell lines (Pan et al., 2020) and regulation of the epithelial-mesenchymal transition (EMT) process by controlling the janus tyrosine kinase 2 (JAK2)/signal transducer and activator of transcription 3 (STAT3) and Src homology region 2 domain-containing phosphatase-2 (SHP2)/growth factor receptor-bound protein 2 (Grb2) pathways in lung cancer (Wang et al., 2022). Additionally, in integrative exome sequencing analysis, SH2B3 showed high mutation rates in castration-resistant prostate cancer (Hao et al., 2020).

In this study, we investigated the novel role of SMYD5 in lung cancer metastasis. The overexpression of SMYD5 in lung cancer cells was found to contribute to cell migration and invasion by directly regulating the expression of the SH2B3 gene through controlled trimethylation of H4K20. Therefore, targeting the activity or expression of SMYD5 using specific inhibitors or siRNAs to reduce cancer metastasis could enhance the effectiveness of lung cancer treatment.

## MATERIALS AND METHODS

### Cell Culture and Reagents

The human lung cancer cell lines NCI-H1299 (lung carcinoma, H1299) and NCI-H1703 (lung squamous cell carcinoma, H1703) were purchased from the Korean Cell Line Bank and cultured in roswell park memorial institute (RPMI) supplemented with 10% fetal bovine serum and 1% penicillin/streptomycin in a humidified atmosphere with 5% CO_2_ at 37 °C. Human TGF-beta 1 (CHO-derived) was purchased from PeproTech (cat no. 100-21C).

### Small interfering RNA Transfection

Small interfering RNA (siRNA) duplexes against SMYD5 (siSMYD5; 5’-GACCGUUGGAUCAG ACUCU-3’, 5’-AGAGUCUGAUCCAACGGUC-3’) and SH2B3 (siSH2B3; 5’-CACAGAUUCCCUUAACCAA-3’, 5’-UUGGUUAAGGGAAUCUGUG-3’) were purchased from Bioneer Co, Ltd. Negative control siRNA (siCont; 5’-AUGAACGUGAAUU GCUCAATT-3’, 5’-UUGAGCAAUUCACGUUCAUTT-3’) was used as a control treatment. The siRNAs were transfected into cancer cell lines using Lipofectamine RNAiMAX (Invitrogen) according to the manufacturer’s protocol. For each well, we used 4 µL transfection agent and a final concentration of 100 nM siRNA. The mixture was mixed gently and incubated for 20 min at room temperature. After treatment, the cells were incubated for 48 h in a 5% CO_2_ incubator at 37 °C.

### Quantitative Real-time Polymerase Chain Reaction

Total RNA was isolated from the indicated cell lines using a Qiagen RNeasy Mini Kit according to the manufacturer’s instructions. RNA aliquots of 1 µg were then reverse transcribed using the iScript cDNA (complementary DNA) synthesis kit (Bio-Rad Laboratories, Inc.) according to standard protocols. For quantitative real-time polymerase chain reaction (RT-PCR), PCR was performed using AriaMx Real-Time PCR (Agilent Technologies) following the manufacturer’s instructions. Quantitative real-time PCR was performed on cDNA samples using Brilliant III Ultra-Fast SYBR Green QPCR Master Mix (Agilent Technologies), and the signal was detected with an AriaMx Real-time PCR System (Agilent Technologies). The fluorescence threshold value was calculated using Agilent Aria 1.6 software. The PCR primers used were as follows: SMYD5 (forward, 5'-TACCCACCTGAG ACTGCAAG-3' and reverse, 5'-TCTCCGCAGAAGTTCCAGTT-3'), SH2B3 (forward, 5'-GTGGGGAATACGTGCTCACTT-3' and reverse, 5'-TGTCCACGACCGAGGGAAA-3'), N-cadherin (forward, 5'-AGCCAACCTTAACTGAGGAGT-3' and reverse, 5'-GGCAAGTTGATTGGAGGGATG-3'), E-cadherin (forward, 5'-ATTTTTCCCTCGACACCCGAT-3' and reverse, 5'-TCCCAGGCGTAGACCAAGA-3'), matrix metalloproteinase-9 (MMP9; forward, 5'-TCCAGTACCGAGAGAAAGCC-3' and reverse, 5'-CATAGGTCACGTAGCCCACT-3'), actin beta (ACTB; forward, 5'-ACTCTTCCAGCCTTCCTTCC-3' and reverse, 5'-CAATGCCA GGGTACATGGTG-3'), Claudin1 (forward, 5'-TGGTCAGGCTCTCTTCACTG-3' and reverse, 5'-TTGGATAGGGCCTTGGTGTT-3').

### Migration and Invasion Assays

Transwell inserts were coated with a 2% gelatin solution and incubated at room temperature for 5 h for the migration assay. The gelatin-coated Transwell inserts (353097, BD Falcon) and invasion chambers (354480, Corning) were rehydrated in serum-free medium. A complete medium with 20% fetal bovine serum (700 µL) served as a chemoattractant in the bottom chamber. Approximately 1 × 10^5^ cells/well were incubated in the plates for 40 h at 37 °C with 5% CO_2_. In addition, during TGF-β treatment, the cells were incubated for 12 h. At the end of the incubation period, the migrated and invaded cells were fixed with methanol for 5 min and stained with 0.1% crystal violet.

### Wound Healing Assay

Cells were seeded in 6-well plates and wounded by scratching with sterile plastic 10 µL micropipette tips after 48 h of siRNA infection, and incubated in a humidified atmosphere with 5% CO_2_ at 37 °C. Then, the cells were washed with phosphate buffer solution (PBS), and fresh serum medium or inhibitor-treated medium was added. The cells were photographed at 0 h and 24 h after wounding via a CELENA S Digital Imaging System (Logos Biosystems). The cell migration distance was observed in the photographs.

### Immunohistochemistry

Paraffin-embedded sections of the human lung tumor tissue array (T8235732-5, BioChain) were processed in a microwave (90 °C) with antigen-retrieval solution (pH 9) (S2367; Dako), treated with a peroxidase-blocking reagent, and then treated with a protein-blocking reagent (K130, X0909; Dako). Tissue sections were incubated with rabbit anti-SMYD5 antibody (A6191; ABclonal) followed by incubation with an horseradish peroxidase (HRP)-conjugated secondary antibody (Dako). Immunoreactivity was visualized with a chromogenic substrate (Liquid DAB Chromogen; Dako). Finally, tissue specimens were stained with Mayer's hematoxylin solution (Hematoxylin QS; Vector Laboratories) for 5 s to discriminate the nucleus from the cytoplasm. Immunochemistry was performed according to a standard protocol.

### Chromatin Immunoprecipitation

Chromatin immunoprecipitation (ChIP) was performed with a Simple ChIP Plus Sonication Chromatin IP Kit (#56383S; CST) following the manufacturer's instructions. NCI-H1299 and H1703 cells transfected with siCont and siSMYD5 for 48 h were crosslinked with 1% formaldehyde (Sigma-Aldrich) for 10 min at room temperature and quenched with 1× glycine for 5 min at room temperature. Then, the cells were washed with cold 1× PBS (containing 1× Protease Inhibitor Cocktail) and lysed in 1× cell lysis buffer (containing 1× Protease Inhibitor Cocktail). Then, after nuclear extraction, the chromatin solution was sonicated using a Bioruptor Pico sonication device (B01060010; Diagenode) with 20 cycles of 30 s ON and 30 s OFF to obtain 200 to 1,000-bp chromatin fragments. Sheared chromatin (approximately 5-10 µg) was incubated with 2 μg of anti-H4K20me3 (ab9053; Abcam) ChIP-grade antibodies at 4 °C (overnight). After overnight incubation, complexes with 30 µL of ChIP-Grade Protein G Magnetic Beads were incubated for 2 h at 4 °C. Then, the complexes were washed for each step, incubated with ChIP elution buffer for 30 min at 65 °C, and then incubated with proteinase K for 2 h at 65 °C. After DNA purification using spin columns, the samples were analyzed by quantitative PCR using SH2B3 primers. The primers were as follows: SH2B3 promoter region (P1) forward, 5’-GCGACTTGTTTCCTGTTGGG-3’ and reverse, 5’-TCATGGGGAGCTGAGACGGA-3’ SH2B3 (P2) forward, 5’-AGAGGGCTTCCTGGAGA AAG-3’ and reverse, 5’-CTTTGCCTTTGCTACAGCCG-3’ SH2B3 (P3) forward, 5’-TCGC AGATAGTCAGGGTCGG-3’ and reverse, 5’-TAGCGACCACGGCTTTCATC-3’.

### RNA-Sequencing Analysis

Using the TrueSeq RNA Sample Preparation Kit V2, purification and library construction were carried out with total RNA, and Illumina NextSeq 1000 machines (Illumina) were used for sequencing with a read length of 2 × 100 bases. A filtered read set was created using the Cutadapt v1.18 (https://cutadapt.readthedocs.io/en/stable/) command line parameters ‘-a AGATCGGAAGAGCACACGTCTGAACTCCAGTCAC -AAGATCGGAA GAGCGTCGTGTAGGGAAAGAGTGTA-m 50-O 5’ and Sickle v1.33 (https://github.com/n ajoshi/sickle) was used to remove the low-quality sequence (Phred score < 20) to a minimum length of 50 bp. We assessed the quality of the paired-end reads using FastQC version 0.11.4. Additionally, duplicate sequences were examined through the application of the FASTQC tool. The trimmed data containing low-quality reads and the poly-N sequences were processed using the NGSQCToolkit v2.3.3 (https://github.com/mjain-lab/NGSQCToolkit). Then reads were aligned to human genome assembly GRCh38.97 (Accession no. GCA_000001405.27) by HISAT2 v2.1.0 (https://daehwankimlab.github.io/hisat2/). Transcripts obtained were quantified in fragments per kilobase million format using StringTie v2.2.1 (https://github.com/gpertea/stringtie) to calculate expression values and obtain normalized counts. Among total genes (*n* = 60,558), protein-coding genes (*n* = 19,957) were selected according to the Ensemble database (https://ensembl.org/Homo_sapiens/).

### Immunocytochemistry

Cells were seeded (H1299 2.5 × 10^4^ cells/well or H1703 5 × 10^4^ cells/well; 4-well chamber slides), treated as described above, and then fixed in 4% formaldehyde at room temperature for 10 min. The endogenous peroxidase activity was blocked with Triton-X100 in PBS blocking solution for 10 min at room temperature. Cells were washed with PBS and incubated with monoclonal SH2B3 antibody (ORIGENE, TA502764) at 4 °C overnight. SH2B3 expression was visualized using Alexa Fluor 488 (green) secondary antibodies (Thermo Fisher, A11029 or A11034). Nuclei (blue) were labeled with VECTASHIELD fixed cell stain (VESTOR, Cat no. H-1200). Slides were then visualized in a CELENA S Digital Imaging System (Logos Biosystems).

### Statistical Analysis

To classify patients into 2 groups, we applied receiver operating characteristic analysis to determine the best cutoff (defined as the point with the highest level of sensitivity and specificity) for the gene expression value of SMYD5. Comparisons between 2 groups were performed using an unpaired *t* test. One-way analysis of variance was followed by Tukey's honest significant difference post hoc test for multiple comparison groups. Significance was determined according to the *P* value provided by the database. The Spearman correlation values indicated were provided by the TCGA database. Significance is indicated as follows: *P < .05; **P < .01; ***P < .001.

## RESULTS

### SMYD5 Knockdown Suppressed the Migration and Invasion of Lung Cancer Cell Lines

**Fig. 1:**
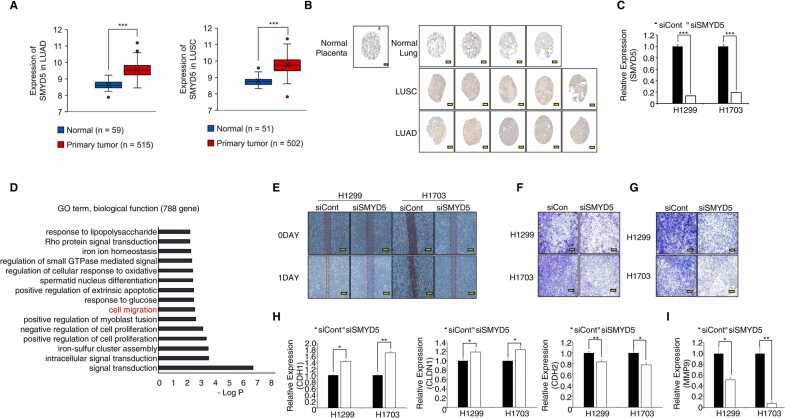
SMYD5 knockdown suppressed mesenchymal function in lung cancer cell lines. (A) SMYD5 expression levels in normal samples versus lung adenocarcinoma (LUAD, left) or lung squamous cell carcinoma (LUSC, right) patient samples derived from the TCGA portal. The mean ± SD of 3 independent experiments is presented. *P* values were calculated using Student’s *t test* (***P < .001). (B) Immunohistochemistry (IHC) staining of SMYD5. Scale bar, 200 µm. (C) The expression of SMYD5 decreased in 2 types of lung cancer cell lines (NCI-H1299 and NCI-H1703) after siSMYD5 treatment, as determined by qRT-PCR analysis. The mean ± SD of 3 independent experiments is presented. *P* values were calculated using Student’s *t test* (***P < .001). (D) Identification of 788 DEGs that overlap DAVID-based gene ontology analysis of RNA-seq results from the siCont versus siSMYD5 groups in NCI-H1299 cell lines. (E) Wound healing assay for analysis of cell migration in NCI-H1299 (H1299, left) and NCI-H1703 (H1703, right) cells after transfection with siSMYD5. Scale bar, 200 µm. (F and G) Transwell assays were performed to investigate the role of SMYD5 in cell migration (left) and invasion (right). The images were obtained 40 h after seeding. Scale bar, 200 µm. (H and I) qRT-PCR analysis of EMT markers (CDH1, CDH2, Claudin-1 (CLND1), MMP9) after SMYD5 siRNA or siCont treatment in NCI-H1299 and H1703 cell lines. ACTB was used as an internal control. The mean ± SD of 3 independent experiments is presented. *P* values were calculated using Student’s *t test* (**P < .01, *P < .05). DEGs, differentially expressed genes.

In the cancer genome atlas program (TCGA) portal, SMYD5 was overexpressed in 2 types of lung cancer (lung adenocarcinoma and lung squamous cell carcinoma) samples compared to normal samples (Fig. 1A). To validate SMYD5 overexpression, we performed immunohistochemistry with an SMYD5 antibody in a tissue microarray. Figure 1B shows that SMYD5 was also overexpressed in lung cancer tissues compared to normal tissues. In the knockdown of SMYD5 mRNA expression levels by siSMYD5 treatment (Fig. 1C). Next, to identify the function of SMYD5 in lung cancer, we performed RNA-sequencing (RNA-seq) analysis after SMYD5 knockdown with SMYD5-specific siRNA and found differentially expressed genes (788 upregulated, 772 downregulated). Using 788 upregulated genes, Gene Ontology (GO) analysis was performed with database for annotation, visualization and integrated discovery (DAVID; https://david.ncifcrf.gov/home.j). In the biological function category, cell migration- or proliferation-related GO terms such as “cell migration” “positive regulation of cell proliferation” and “extrinsic apoptotic signaling pathway” were enriched by SMYD5 knockdown in NCI-H1299 cell lines (Fig. 1D). Moreover, the “regulation of actin filament organization” term was enriched in GO analysis according to the ClueGO plugin of Cytoscape ver 3.7.1 (data not shown). Thus, we suggest that SMYD5 may regulate cell migration or invasion to facilitate lung cancer metastasis. To validate the function of SMYD5 in lung cancer metastasis, we performed cell wound healing analysis after SMYD5 knockdown in NCI-H1299 and H1703 cell lines. The recovery rate of wound healing was decreased compared to that in siControl (siCont)-transfected NCI-H1299 and H1703 cell lines (Fig. 1E). Moreover, cell migration and invasion analyses showed that the number of migrated or invaded cells was significantly decreased by siSMYD5 treatment (Fig. 1F and G). In terms of EMT markers, the levels of CDH1 (E-cadherin) and CLDN1 (epithelial markers) were increased, and the expression of CDH2 (a mesenchymal marker, N-cadherin) was decreased in the SMYD5 knockdown group compared to the siCont group (Fig. 1H). In the invasion assay, since cell invasion was suppressed by SMYD5 knockdown, we assessed MMP9 expression after the knockdown of SMYD5 and found that MMP9 expression was decreased in the SMYD5 knockdown group compared to the siCont group of NCI-H1299 and H1703 cell lines (Fig. 1I). Taken together, these results suggest that SMYD5 regulates migration and invasion in lung cancer.

### SH2B3 is a Direct Target of SMYD5 in Lung Cancer Metastasis

**Fig. 2:**
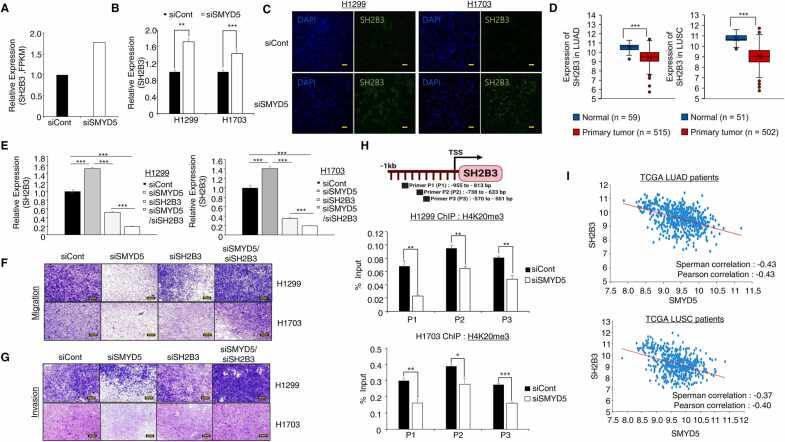
SH2B3 is directly regulated by SMYD5-related epigenetic regulation. (A) RNA-seq analysis of SH2B3 expression levels after treatment with SMYD5 siRNA versus siCont in NCI-H1299 cell lines. (B) qRT-PCR analysis of SH2B3 expression levels after SMYD5 siRNA or siCont treatment in NCI-H1299 (H1299) and NCI-H1703 (H1703) cell lines. The mean ± SD of 3 independent experiments is presented. *P* values were calculated using Student’s *t test* (***P < .001, **P < .01). (C) Immunocytochemical analysis of SH2B3 after SMYD5 knockdown. H1299 and H1703 cells treated with siCont, siSMYD5 were fixed with 100% methanol and stained with an anti-SH2B3 antibody (Alexa Fluor 488, green) and DAPI (4',6-diamidino-2-phenylindole; blue). Scale bar, 200 µm. (D) Boxplot showing the relative expression of SH2B3 in normal versus tumor tissues. Lung adenocarcinoma (LUAD, left) and lung squamous cell carcinoma (LUSC, right) samples were derived from the TCGA portal. (E) qRT-PCR analysis of target gene expression levels (SH2B3) after SMYD5/SH2B3 coknockdown in NCI-H1299 and NCI-H1703 cells. The mean ± SD of 3 independent experiments is presented. Statistical analysis was performed by 1-way ANOVA followed by Tukey's honest significant difference (HSD) post hoc test for multiple comparisons (***P < .001*).* (F and G) Migration (upper) and invasion (lower) assays after SMYD5/SH2B3 coknockdown in NCI-H1299 and NCI-H1703 cells. Cell migration and invasion assays were performed after 40 h. Migrated/invaded cells were stained with crystal violet. Scale bar, 200 µm. (H) Graphical abstract for ChIP primer design on the SH2B3 promoter region. ChIP assays were performed using an anti-H4K20 trimethylation antibody on the SH2B3 promoter region. The levels of H4K20me3 in the SH2B3 gene were determined by ChIP-qPCR. Data are the average of three independent replicates. The mean ± SD of 3 independent experiments is presented. *P* values were calculated using Student’s *t test* (***P < .001, **P < .01, *P < .05). (I) Gene expression correlation analysis for SMYD5 with the SH2B3 gene in LUAD (upper) and LUSC (lower) samples derived from the TCGA portal. ANOVA, analysis of variance.

SMYD5 can methylate H3K36 and H4K20 trimethylation for upregulation and downregulation of gene expression (Kidder et al., 2017, Zhang et al., 2022). Histone H3K36 dimethylation mainly involves the upregulation of gene expression in the gene body region (Huang and Zhu, 2018), but histone H4K20 trimethylation is associated with the formation of heterochromatin structures in the promoter region of the gene for the downregulation of gene expression (Brustel et al., 2017). Additionally, Stender et al. (2012) showed that overexpression of SMYD5 led to an increase in the amount of H4K20 trimethylation, while the levels of other marks, such as H3K36me3, H3K27me3, H3K9me3, and H3K4me3, remained unchanged. Thus, in this study, to identify metastasis-regulated genes in lung cancer, we focused on genes upregulated by SMYD5 knockdown. Finally, we selected the upregulated SH2B3 gene and assessed its role in lung cancer metastasis because, in lung cancer cell lines, overexpression of SH2B3 decreased the number of migrated cells and the wound healing rate and induced E-cadherin expression (Wang et al., 2022). In the RNA-seq results, the expression of SH2B3 was increased in the SMYD5 knockdown group compared to the siCont group (Fig. 2A). We confirmed the upregulation of SH2B3 by SMYD5 knockdown via qRT-PCR analysis in NCI-H1299 and H1703 cell lines (Fig. 2B). Moreover, we observed the upregulation of SH2B3 expression by SMYD5 knockdown in immunocytochemistry (Fig. 2C). In the TCGA data portal, the expression of SH2B3 was decreased in lung cancer compared to normal samples (Fig. 2D). To validate that the SMYD5-SH2B3 axis represses migration and invasion, we performed rescue analysis after cotreatment with siSMYD5 and siSH2B3 and observed the regulation of SH2B3 expression by siSMYD5 and siSH2B3 in qRT-PCR analysis (Fig. 2E). In migration and invasion assays, the reduction in the number of migratory and invasive cells upon SMYD5 knockdown was recovered after cotreatment with siSMYD5 and siSH2B3 (Fig. 2F and G), suggesting that SMYD5 knockdown upregulated the expression of SH2B3 to reduce cell migration and invasion. To verify the direct target of SMYD5 in SH2B3 cells, we performed a ChIP assay after SMYD5 knockdown with an anti-H4K20 trimethylation antibody. We designed ChIP primers on the promoter region of SH2B3 (Fig. 2H, upper) and found a reduction in the H4K20 trimethylation status by SMYD5 knockdown (Fig. 2H, lower), implying that SH2B3 is a direct target for SMYD5 in NCI-H1299 and H1703 cell lines. Therefore, utilizing a cotransfection system, we propose that the SMYD5 knockdown-induced upregulation of SH2B3 expression using siSH2B3/siSMYD5 resulted in reduced migration and invasion compared to the transfection of SMYD5 siRNA alone. In other words, the decrease in cell migration and invasion observed with SMYD5 knockdown was linked to the increased expression of SH2B3 in lung cancer. Moreover, in the TCGA portal, we observed a negative correlation between SMYD5 and SH2B3 (Fig. 2I). Thus, we suggest that SMYD5 is involved in lung cancer metastasis via the critical regulation of SH2B3 expression.

### SMYD5 Knockdown Suppressed Cell Migration and Invasion in the TGF-β-induced In Vitro EMT System

**Fig. 3:**
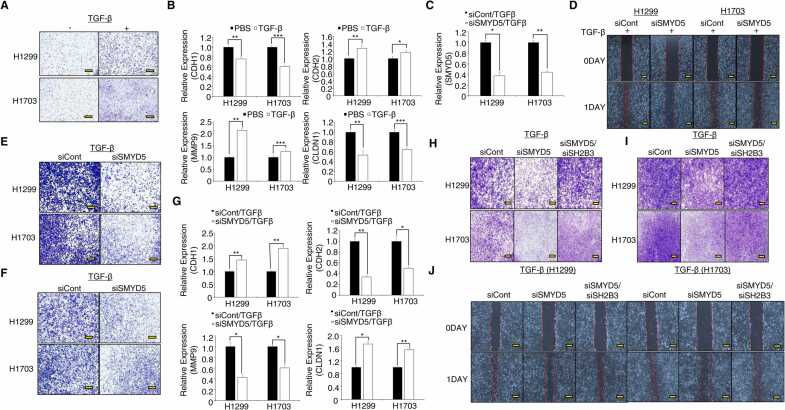
SMYD5 is a key regulator of lung cancer cell behaviors. (A) Wound healing analysis of cell migration in NCI-H1299 (H1299, upper) and NCI-H1703 (H1703, lower) cells after TGF-β treatment for 24 h. Scale bar, 200 µm. (B) qRT-PCR analysis of EMT markers (N-, E-cadherin, Claudin-1 (CLND1), MMP9) after TGF-β treatment in NCI-H1299 and H1703 cell lines. ACTB was used as an internal control. The mean ± SD of 3 independent experiments is presented. *P* values were calculated using Student’s *t* test (***P < .001, **P < .01, *P < .05). (C) The expression of SMYD5 decreased in 2 types of lung cancer cell lines (NCI-H1299 and NCI-H1703) after siSMYD5 treatment with 5 ng/mL TGF-β for 24 h, as determined by qRT-PCR analysis. The mean ± SD of 3 independent experiments is presented. *P* values were calculated using Student’s *t* test (**P < .01, *P < .05). (D) Wound healing analysis of cell migration in NCI-H1299 (left) and NCI-H1703 (right) cells after transfection with siSMYD5 and 5 ng/mL TGF-β for 24 h. Scale bar, 200 µm. Transwell assays were performed to investigate the role of SMYD5 with TGF-β treatment hours in cell migration (E) and invasion (F). The images were obtained 12 h after seeding. Scale bar, 200 µm. (G) qRT-PCR analysis of EMT markers (CDH1, CDH2, Claudin-1 (CLND1), MMP9) after SMYD5 siRNA with TGF-β treatment in NCI-H1299 and H1703 cell lines. ACTB was used as an internal control. The mean ± SD of 3 independent experiments is presented. *P* values were calculated using Student’s *t* test (**P < .01, *P < .05). Migration (H) and invasion (I) assays after SMYD5/SH2B3 coknockdown with TGF-β treatment in NCI-H1299 and NCI-H1703 cells. Cell migration and invasion assays were performed after 12 h. Migrated/invaded cells were stained with crystal violet. Scale bar, 200 µm. (J) Wound healing assay for analysis of cell migration in NCI-H1299 (left) and NCI-H1703 (right) cells after SMYD5/SH2B3 coknockdown with TGF-β treatment. Scale bar, 200 µm.

Transforming growth factor-beta (TGF-β) treatment can induce in vitro EMT in cancer cell lines (Al Ameri et al., 2019). Thus, we tried to construct an in vitro EMT system to confirm SMYD5 function in highly invasive cell lines. After treatment of NCI-H1299 and H1703 cell lines with TGF-β, we observed increased cell migration and wound healing assays and found altered EMT markers, suggesting that we successfully constructed an in vitro EMT system (Fig. 3A and B). To verify whether SMYD5 knockdown could affect cell migration and invasion in an in vitro EMT system, we added siSMYD5 to TGF-β-treated cell lines and confirmed SMYD5 knockdown by siSMYD5 treatment in highly invasive cell lines (Fig. 3C). Wound healing and migration analysis showed that SMYD5 knockdown also decreased the wound healing rate and number of migrated cells in highly invasive cell lines (Fig. 3D and E). Additionally, we observed a reduction in the number of invasive cells among SMYD5 knockdown cells compared to siCont cells, implying that SMYD5 also regulates migration and invasion in highly invasive cell lines (Fig. 3F). Moreover, we found changes in EMT markers and MMP9 expression upon SMYD5 knockdown in highly invasive cell lines (Fig. 3G). Next, in highly invasive cell lines, the reductions in cell migration, invasion, and wound healing rate by SMYD5 knockdown were attenuated after cotransfection of siSMYD5 and siSH2B3 (Fig. 3H-J). Thus, SMYD5 may be an important regulator of lung cancer metastasis.

## DISCUSSION

SMYD5 is a methyltransferase that facilitates the reconstruction of chromatin structures, such as euchromatin and heterochromatin structures via histone methylation (Kidder et al., 2017b). In the self-renewal and differentiation of ES cells, SMYD5 knockdown reduced the global H4K20 trimethylation level and upregulated lineage-specific genes. However, SMYD5 also facilitates H3K36 and K37 monomethylation in murine ES cells (Aljazi et al., 2022) and H3K36 trimethylation at the gene promoter region for the positive regulation of gene expression (Zhang et al., 2022). In this study, to identify the direct target by which SMYD5 facilitates lung cancer metastasis, we focused on 788 upregulated genes in the RNA-seq results after SMYD5 knockdown because SMYD5 mainly trimethylates H4K20 to repress gene expression via heterochromatin construction. However, because SMYD5 also methylates H3K36 and H3K37 in the gene promoter region, the 772 genes downregulated by SMYD5 knockdown may be involved in lung cancer metastasis. Thus, in further studies, ChIP-seq and ATAC-seq analyses will be performed to verify metastasis-related genes related to SMYD5 in the context of lung cancer metastasis.

In the GO term analysis using RNA-seq results, proliferation- and apoptosis-related terms were enriched in the SMYD5 knockdown group compared to the siCont group (Fig. 1D). However, in this study, we could not detect growth suppression by SMYD5 knockdown in NCI-H1299 and H1703 cell lines (data not shown), but SMYD5 knockdown reduced cell migration, invasion, and wound healing rates in lung cancer cell lines, implying that the function of SMYD5 in lung cancer is mainly involved in lung cancer metastasis. Recently, we reported that SMYD2 knockdown reduced lung cancer metastasis in vitro and in vivo metastasis analyses, but similar to SMYD5 knockdown, SMYD2 knockdown did not affect cell growth inhibition in lung cancer cell lines (Kim et al., 2023). Therefore, our results suggest that SMYD family members (specifically SMYD2 and SMYD5) are important factors in lung cancer metastasis.

The main cause of lung cancer-related death is metastasis. The common sites of lung cancer metastasis are bone, liver, brain, lymph nodes, and adrenal glands (Riihimaki et al., 2014). During anticancer treatment in lung cancer, to reduce the incidence of distant metastasis and micrometastasis, cotreatment with SMYD5-specific inhibitors and general anticancer drugs is needed. In this study, SMYD5 knockdown clearly suppressed TGF-β-induced migration and invasion of highly invasive lung cancer cell lines (Fig. 3E and F). Thus, to increase the efficiency of lung cancer treatment, SMYD5-specific inhibitors need to be developed.

**Fig. 4:**
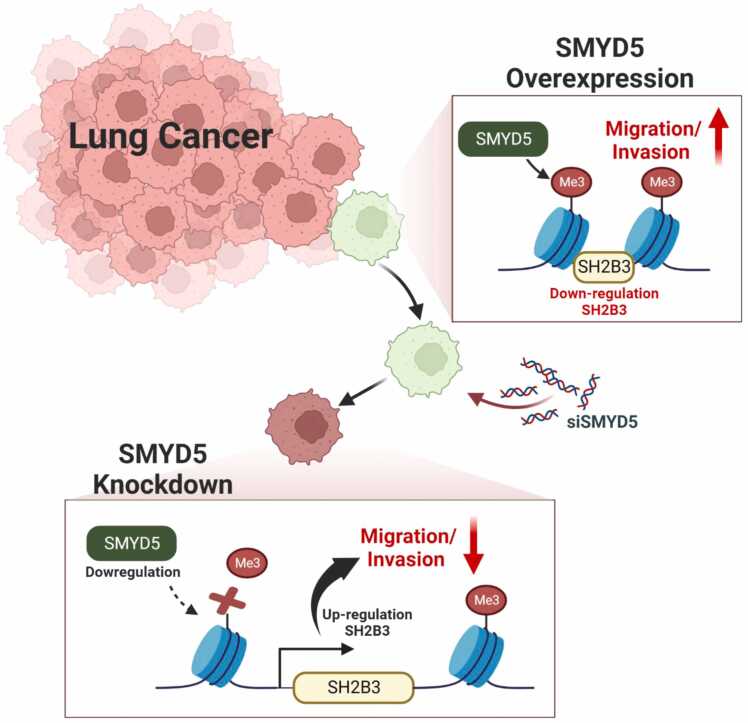
Schematic summary model.

In conclusion, we identified SMYD5 overexpression in lung cancer with TCGA data portal and immunohistochemical analysis. Knockdown of SMYD5 clearly affected the EMT process and suppressed cell migration and invasion via upregulation of SH2B3 (Fig. 4). Moreover, we confirmed SH2B3 as a direct target of SMYD5 using a ChIP assay. Thus, we suggest that direct targeting of SMYD5 with siRNA or specific inhibitors for the repression of lung cancer metastasis is an important strategy for lung cancer treatment. Thus, SMYD5-specific inhibitors should be established in further studies.

## Author contributions

H-S.C., D-S.K., and M-Y.S.: conception and design. I.H.T., T.Y.R., Y.K., J.L., K.K., J.M.L., and H-W.K.: development of methodology. H-W.K., J.H.K.: analysis and interpretation of data. J.H.K., D-S.K., M-Y.S., and H-S.C.: writing and review of manuscript, study supervision.

## Declaration of Competing Interests

The authors have no financial conflicts of interest to declare.
